# Innate Lymphoid Cells: Expression of PD-1 and Other Checkpoints in Normal and Pathological Conditions

**DOI:** 10.3389/fimmu.2019.00910

**Published:** 2019-04-26

**Authors:** Francesca Romana Mariotti, Linda Quatrini, Enrico Munari, Paola Vacca, Lorenzo Moretta

**Affiliations:** ^1^Department of Immunology, IRCSS Bambino Gesù Children's Hospital, Rome, Italy; ^2^Department of Pathology, Sacro Cuore Don Calabria Hospital, Negrar, Italy

**Keywords:** NK, ILC, PD-1, checkpoint receptors, cancer immunotherapies

## Abstract

Innate lymphoid cells (ILCs) belong to a family of immune cells. Recently, ILCs have been classified into five different groups that mirror the function of adaptive T cell subsets counterparts. In particular, NK cells mirror CD8^+^ cytotoxic T cells while ILC1, ILC2, ILC3, and Lymphoid tissue inducer (LTi)-like cells reflect the function of CD4^+^T helper (Th) cells (Th1, Th2, and Th17 respectively). ILCs are involved in innate host defenses against pathogens and tumors, in lymphoid organogenesis, and in tissue remodeling/repair. In recent years, important molecular inducible checkpoints (PD-1, TIM3, and TIGIT) were shown to control/inactivate different immune cell types. The expression of many of these receptors has been detected on NK cells and subsets of tissue-resident ILCs in both physiological and pathological conditions, including cancer. In particular, it has been demonstrated that the interaction between PD-1^+^ immune cells and PD-L1/PD-L2+ tumor cells may compromise the anti-tumor effector function leading to tumor immune escape. However, while the effector function of NK cells in tumor is well-established, limited information exists on the other ILC subsets. We will summarize what is known to date on the expression and function of these checkpoint receptors on NK cells and ILCs, with a particular focus on the recent data that reveal an essential contribution of the blockade of PD-1 and TIGIT on NK cells to the immunotherapy of cancer. A better information regarding the presence and the function of different ILCs and of the inhibitory checkpoints in pathological conditions may offer important clues for the development of new immune therapeutic strategies.

## Introduction

Innate Lymphoid Cells (ILCs) represent a heterogeneous group of developmentally related lymphocytes (1). However, distinct aspects differentiate T or B lymphocytes from ILCs. Thus, unlike T and B cells, ILCs are characterized by the lack of expression of recombination activating genes (RAG-1 and RAG-2)-dependent rearranged antigen receptors and rely on a set of germ-line encoded receptors to exert their function (2–5). Thus, while T cells responses require longer time intervals due to antigen-mediated clonal selection and expansion, ILCs can exert a prompt response to recognition of conserved molecular patterns from pathogens and infected or injured tissues (6). For this reason and for their tissue-residency ILCs may represent the leading orchestrator of immune responses. ILCs release effector and regulatory cytokines that play a role in tissue repair and immune defense being also able to coordinate the adaptive immune responses (7–9). Thus, ILCs might also play a primary role in sensing cells that underwent malignant transformation and in initiating anti-tumor immune response, even though, as will be discussed in this review, their actual role in tumor suppression is controversial.

## ILC Differentiation and Classification

**Figure 1:**
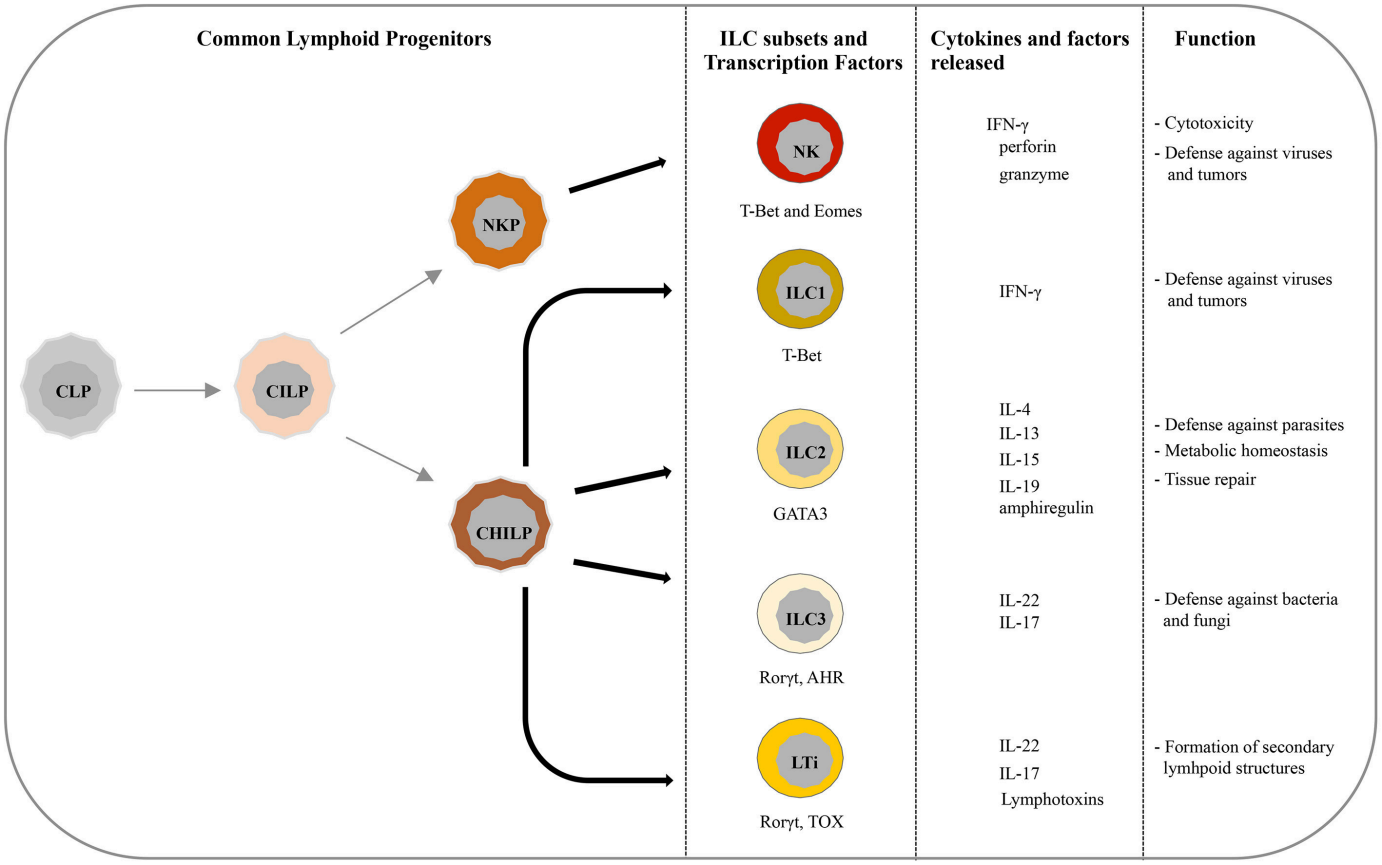
Current view of differentiation and immune function of ILC subsets. ILCs originate from common lymphoid progenitors (CLPs) that give rise to common innate lymphoid progenitors (CILPs). On one side, CILPs can differentiate into NK precursor (NKP) that, through T-bet and Eomes, will originate NK cells, and on the other side into common helper innate lymphoid precursors (CHILP) from which originate both Innate lymphoid precursors (ILP) and lymphoid tissue inducer progenitors (LTiP). ILC1/2/3 subsets and LTi cells differentiate, according to the different transcription factors required, from ILP and LTiP precursors, respectively. As specified in the text, NK/ILC cells release various cytokines and exert different immune functions.

Recently, five main ILCs groups have been identified according to the transcription factors required for their development and function (6, 10). These groups are represented by natural killer cells (NK), group 1 ILCs (ILC1), group 2 ILCs (ILC2), group 3 ILCs (ILC3), and lymphoid tissue-inducer (LTi) cells. All ILCs derive from common lymphoid progenitors (CLPs) that mainly reside in the bone marrow, even though ILCs progenitors are also found in other tissues, such as fetal liver, tonsils, decidua, and intestinal lamina propria (9, 11–16). ILCs are divided into cytotoxic-ILC and helper-ILC, which resemble cytotoxic and helper T cell subsets (17). In particular, NK cells mirror cytotoxic CD8^+^ cells while ILC1, ILC2, and ILC3 resemble the T helper cell subsets Th1, Th2, and Th17, respectively. Originally NK and ILC1 represented the group 1 of ILC family because both subsets express the transcription factor T-bet, and secrete interferon (IFN)-γ and tumor necrosis factor (TNF)-α (18, 19) (Figure 1). However, it must be noticed that NK cells, apart from T-bet, rely also on Eomes expression for their development (20) (Figure 1). NK cells, the first ILC subset to be identified, circulate in the bloodstream where they represent about the 15% of peripheral blood lymphocytes (1, 21). However, tissue-resident NK cells have also been found in liver, uterus and decidua (15, 22–24). In humans, two main PB-NK cell subsets can be distinguished based on the level of CD56 surface expression (25). In particular CD56^dim^, expressing high levels of perforin and granzyme, are characterized by a high cytotoxic activity, while CD56^bright^ cells secrete inflammatory cytokines and are prevalently found in tissues and secondary lymphoid organs (26). NK cells play a major role in innate defenses against viruses and tumors, both by direct cell killing and by promoting the initiation of inflammation (27–30). On the contrary, ILC1 express low level of perforin and are barely found in PB while they mainly reside within tissues, such as intestine, lung, skin and decidua where they are involved in the first line of defense against viruses and bacteria (19, 31–33). ILC2s, which rely on GATA3 expression, are mostly found in lung, intestine, adipose tissue, skin, and gut (34). Upon activation in response to epithelia-derived stimuli (mainly IL-33 and IL-25), they release type-2 cytokines (primarily IL-5, IL-13, and IL-9) and promote defenses against parasites, viral infections, and contribute to metabolic homeostasis (7, 31, 35, 36). In addition, ILC2s produce amphiregulin, an epidermal growth factor family member involved in tissue repair (17). The most heterogeneous ILC subset includes fetal LTi and postnatal ILC3, both depending on the Rorγt transcription factor. They were previously called “group 3 ILCs” but, because of their different developmental trajectories, they are now classified into two distinct subsets (1). LTi cells play a pivotal role in the formation of secondary lymphoid structures, including lymph nodes and Peyer's patches during fetus development (37, 38). After birth, ILC3s can be found in gut, tonsils, and intestine where, through release of IL-22, play an important role in the innate immunity against bacteria and fungi (38, 39). However, LTi and a particular subset of ILC3, namely NCR^+^ ILC3, have also been found in decidual tissue during early pregnancy (33).

While the role of NK cells in anti-tumor immunity has been widely studied and well-established, ILCs function in the immune defenses against tumors is still controversial (40). Their preferential localization at the mucosal surfaces may even suggest a negative role, as some of their cytokines exacerbate the development of chronic inflammation and potentially favor tumor growth. Indeed, IFN-γ released by ILC1 in inflamed conditions could have detrimental effects by favoring tumor growth. Similarly, type-2 cytokines produced by ILC2s are associated with poor prognosis in cancer patients and can create a pro-tumorigenic environment through the stimulation of myeloid derived suppressor cells (MDSC) or M2 macrophages (2, 41, 42). Moreover, ILC3s have been associated with tumor growth and metastasis in different type of cancers (10). However, available evidence suggests that ILCs function may depend on the tumor microenvironment (43–48). Indeed, the different cytokines, soluble factors, and cell types that characterize the tumor microenvironment can shape the function of different immune cells, dampening antitumor immunity (7). In this context, ILCs plasticity, that allows them to convert from one subset into another depending on the surrounding stimuli, might have a negative role in immune defenses. Therefore, a deeper understanding of NK and ILCs in protective immunity and how tumor cells and the tumor microenvironment can inhibit their functions is of extreme interest especially for the development of new immunotherapies.

## NK/ILC Cell Receptors

NK/ILC are able to discriminate between healthy and virus- or tumor- infected cells through an array of inhibitory and activating receptors that recognize specific ligands induced by virus infection or tumor transformation (49–51). Natural Cytotoxicity Receptors (NCR), which include NKp46, NKp44, and NKp30, represent the major NK cells activating receptors (52–57). NCRs can be also expressed by specific ILCs subsets, with ILC1 expressing NKp46, ILC2 NKp30 and tonsil-derived ILC3 and mucosal NCR^+^ ILC3 bearing NKp30 and NKp46, respectively (58). NKG2D and DNAM-1 represent other important activating receptors able to recognize ligands that are *de novo* expressed or upregulated upon cell stress or tumor transformation (59–62). Additionally, NK cells express co-activating receptors, such as NTB-A and 2B4, whose function depends on the simultaneous co-engagement of one or more activating receptors (57, 63–65). The function of activating receptors is counterbalanced by inhibitory receptors that are mainly represented by the killer Ig-like receptors (KIR) and the heterodimer CD94/NKG2A which recognize the main type of HLA class-I molecules and function as true checkpoints in NK cell activation (29, 66–68). Indeed, in normal conditions these inhibitory receptors recognize HLA-I ligands expressed on healthy cells preventing their killing. As a consequence, loss of MHC expression on tumor cells is increasing rather than decreasing their susceptibility to NK cell-mediated killing (69). Recently, additional inhibitory checkpoints (such as PD-1, TIGIT, etc.), which under normal conditions maintain immune cell homeostasis, have been shown to facilitate tumor escape. Indeed, different studies demonstrated that, in these pathological conditions, checkpoint regulators, usually absent on resting NK cells, can be induced *de novo* and contribute to the downregulation of NK cell anti-tumor function upon interaction with their ligands expressed at the tumor cell surface (70).

In the next paragraphs, we will summarize what is known to date about the expression and function of these checkpoint receptors on NK cells and ILCs, with a particular focus on PD-1, TIGIT, and CD96.

## PD-1

**Figure 2:**
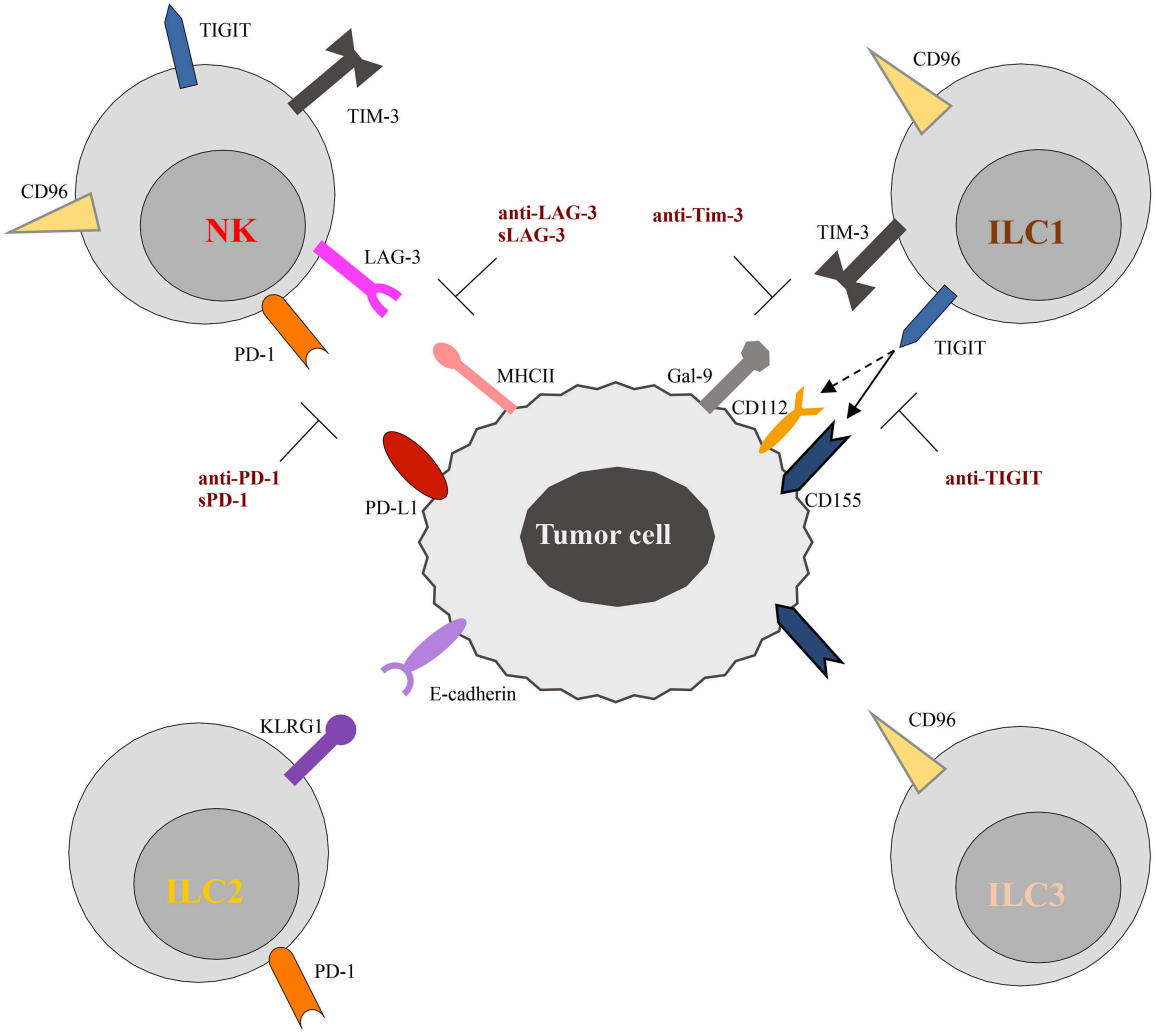
Schematic representation of checkpoint receptors and their ligands expressed by ILC and tumor cells, respectively. NK cells express multiple immune checkpoint receptors, such as PD-1, TIM-3, Lag-3, TIGIT, and CD96. ON the other hand, these checkpoint receptors are instead differentially expressed by ILC subsets. Thus, TIGIT and TIM-3 have been detected only on ILC1 cells, while CD96 is expressed on both ILC1 and ILC2. Surface expression of KLRG1 and PD-1 appears to be restricted to ILC2 cells. The inhibitory ligands expressed by tumor cells, specifically interact with the checkpoint receptors preventing cells activation. However, different therapeutic approaches, aimed to block receptor/ligand interactions, have been demonstrated to restore the anti-tumor activity of immune cells, as illustrated in the Figure. The solid and dotted arrows indicate the strong and weak binding affinity of TIGIT for the different ligands, respectively.

PD-1, a member of immunoglobulin superfamily, is a cell surface inhibitory receptor, functioning as a major checkpoint of T cell activation. It binds PD-L1 and PD-L2, ligands expressed on many tumors, on infected cells, on antigen-presenting cells in inflammatory foci, and in secondary lymphoid organs. Lack of PD-1 expression results in the suppression of tumor growth and metastasis in mice (71). The efficacy of PD-1 blockade has been mainly correlated with the restoration of a preexisting T cell response. PD-1 expression, initially described on T, B, and myeloid cells, has been recently described also on NK cells (72, 73) (Figure 2). In particular, PD-1 expression was shown on NK cells from some healthy individuals and in most cancer patients, including Kaposi sarcoma, ovarian and lung carcinoma and Hodgkin lymphoma, where it can negatively regulate NK cell function (73–78). The contribution of PD-1 blockade on NK cells in immunotherapy has been demonstrated in several mouse models of cancer, where PD-1 engagement by PD-L1^+^ tumor cells could strongly suppress NK cell–mediated anti-tumor immunity (79). PD-1 expression was found more abundant on NK cells with an activated and more responsive phenotype rather than on NK cells with an exhausted phenotype (79). However, to date the molecular mechanisms regulating the expression of this inhibitory receptor on NK cells are not clear. It has been demonstrated in a mouse model of cytomegalovirus infection (MCMV) that endogenous glucocorticoids integrate the signals from the microenvironment to induce PD-1 expression at the transcriptional level, highlighting the importance of a tissue-specific cooperation of cytokines and the neuroendocrine system in this regulation (80). Regarding the cancer setting, however, recent data suggest that PD-1 is accumulated inside NK cells and translocated on the cell surface rather than induced at the transcriptional level (81). However, the stimuli required for its surface expression are unknown.

Two recent papers described that, in mice, PD-1 expression identifies ILC committed progenitors, capable of generating ILC1s, ILC2s, ILC3s, and a small number of circulating NK cells (82, 83). High expression of PD-1 is lost upon differentiation, but upregulated on effector tissue resident ILC2s upon lung inflammation (83). In agreement with these findings, it was shown that mouse ILC2s express PD-1 in different percentages depending on their tissue origin and that this expression is enhanced by IL-33 stimulation, reducing their ability to release cytokines (84). This is particularly relevant in type 2 infections, such as helminth infection, but the role of PD-1 expression on ILC2s in the context of cancer remains to be investigated. Nonetheless, the finding that it is possible to modulate ILC2s effector functions by using PD-1 blocking antibodies suggests that targeting this receptor with checkpoint inhibitors could also affect type 2 responses in cancer patients and favor cancer growth by restoring the production of type-2 cytokines. The possible unfavorable effect of this ILC2-mediated response and its contribution to therapy with checkpoint inhibitors should be further explored to further improve the efficacy of cancer treatment.

Recent studies provided the first evidence that also ILC3s can express a functional PD-1 receptor. In particular, PD-1 expression has been detected on both NK cells and ILC3s in malignant pleural effusions of patients with primary and metastatic tumors (85). Moreover, it has been shown that NK cells and ILC3s in human decidua express PD-1 during the first trimester of pregnancy, while the invading trophoblast expresses PD-L1. The PD-1/PD-L1 molecular interaction regulates ILC3 production of cytokines, suggesting that it may play a regulatory role at the feto-maternal interface (16).

## TIGIT and CD96

TIGIT (T cell Ig and ITIM domain) and CD96 are co-inhibitory receptors expressed on subsets of T cells, human NK cells, ILC1s, and ILC3s (Figure 2). They belong to a group of immunoglobulin superfamily receptors comprising also the co-stimulatory receptor DNAM-1 (CD226). They recognize nectin and nectin-like ligands, frequently upregulated on tumor cells. CD155 is a ligand shared by the three receptors, with CD96 showing the highest binding affinity (86). These receptors initiate a pathway that is analogous to the CD28/CTLA-4 one. In this pathway, ligands and differential receptor:ligand affinities can fine-tune the immune response. The work of Zhang et al. (87) recently demonstrated that TIGIT constitutes a previously unappreciated checkpoint in NK cells, and that targeting TIGIT alone or in combination with other checkpoint receptors may represent a promising anti-cancer therapeutic strategy. It has been shown that, in patients with colon cancer, TIGIT expression is increased on tumor associated NK cells. In addition, evidences has been provided that, beyond the targeting of effector and regulatory T cells, the mode of action of TIGIT blockade also involves NK cells (87). In particular, genetic KO or mAb-mediated blockade of TIGIT was able to unleash both NK cell and T cell antitumor activity, leading to a substantial improvement in the control of tumor growth in several preclinical mouse models. Moreover, TIGIT blockade prevented NK cell exhaustion in the absence of adaptive immunity, and elicited a potent T cell–mediated memory response to tumor re-challenge through a not yet identified mechanism (87). Increased TIGIT and CD96 expression and lower levels of DNAM1 were also detected on ILC1s induced by TGF-β, contributing to the impairment of their anti-tumor response (88).

Although the role of TIGIT and CD96 as immune checkpoint receptors are just beginning to be uncovered, accumulating data would support the notion that targeting of these receptors for improving anti-tumor immune responses also involves NK cells and ILCs.

## Other Checkpoints on NK Cells and ILCs

KLRG1 is another inhibitory receptor expressed by mature NK cells whose expression varies with cell activation. It is a C-type lectin-like receptor containing one ITIM, and it has been used as a marker for distinct NK and T-cell differentiation stages (89). However, KLRG1 knock-out mice showed that it does not play a deterministic role in the generation and functional characteristics of these lymphocyte subsets. KLRG1 is also expressed by mast cells, basophils, eosinophils, and ILC2s, suggesting a role in type 2 immune responses. Experiments in mice showed that *in vivo* administration of IL-25 elicits the expansion of a subset of ILC2s referred to as “inflammatory” ILC2s that are characterized by high expression of KLRG1 and that participate in the control of helminth infection (90). In the tumor context, KLRG1 expression was found on ILC2 associated to the tumor in NSCLC and CRC (91, 92). While the interaction of KLRG1 and its E-cadherin ligand has been shown to inhibit human ILC2s *in vitro*, its function *in vivo* remains to be established (90, 93).

Lag-3 and Tim-3 are inhibitory receptors whose expression has been reported on NK cells and ILC1s (Figure 2). Tim-3 is a type 1 glycoprotein expressed by mature NK cells, and its expression is further increased on NK cells in melanoma and lung adenocarcinoma patients, impairing NK cell effector functions (94, 95). More recently, Tim-3 expression has been reported on human decidual NK cells and also on ILC3s. It was demonstrated that Tim-3 is expressed in higher percentages in CD56^+^ILC3s compared to LTi-like cells, and that its triggering is able to significantly reduce IL-22 production by CD56^+^ILC3s (16). Lag-3 is a negative costimulatory receptor that is homologous to CD4 and binds MHC-II molecules with very high affinity. Although its role in downregulating T cell proliferation, activation, and homeostasis is clear, its mechanism of action in NK cells remains to be dissected in detail (96).

NKG2A is a HLA-E-specific inhibitory receptor that plays an important regulatory role in NK cell function. Also antigen- or cytokine-stimulated T cells were shown to express a functional NKG2A that may antagonize T cell function (97, 98). It has recently been reported that NKG2A is expressed on NK and T cells in the tumor bed in many human cancers such as squamous cell carcinoma of the head and neck (SCCHN) and colorectal carcinoma (CRC) (99). In addition, its ligand, HLA-E, is frequently overexpressed in these tumors. NKG2A targeting with monalizumab (a humanized anti-NKG2A antibody) has been shown to enhance the anti-tumor immunity mediated by NK and CD8^+^ T cells when used as a single agent or in combination with other therapeutic antibodies such as durvalumab (blocking PD-L1), or cetuximab (directed against the epidermal growth factor receptor, EGFR) (99, 100).

In mouse tumor models, it has been shown that TGF-β signaling in the microenvironment induces NK cell conversion to ILC1s. These tumor-associated ILC1s express higher levels of inhibitory receptors (NKG2A, KLRG1, CTLA4, LAG3) as compared to NK cells. While NK cells favored tumor immune surveillance in this setting, the higher expression of immunological checkpoint receptors on ILC1s was associated with a lower ability to control local tumor growth and metastasis (88). These evidences suggest that NK cell conversion to ILC1s displaying a functional impairment could represent an additional mechanism by which tumor escapes immune surveillance.

## Cancer Immunotherapies

During the past few years, different strategies have been developed to overcome the immunosuppressive tumor environment and restore antitumor immune activity. The use of blocking antibodies against inhibitory receptors or their ligands, in order to restore the T or NK cell function has been demonstrated to be an efficient and safe cancer immunotherapy in the treatment of several tumors (70). Considering the wide expression of PD-1 on immune cells, most therapies have been developed in order to block PD-1/PD-L1/2 interactions. Indeed, some anti-PD-1 mAbs have already been approved by FDA, showing encouraging results in patients with melanoma or lung cancer (101, 102). Currently, there are ongoing phase I and II clinical trials for anti-KIR, -NKG2A, -Tim3, -LAG3, -TIGIT inhibitory receptors (102). Interestingly, considering that checkpoint inhibitors can act in synergy with each other, combinations of mAbs are also under investigation as a new approach for optimal boost of the immune system. In particular, clinical trials are investigating the combination of anti-PD1 therapy with anti-TIM3 or anti-TIGIT blocking antibodies in different tumors (70, 103). Moreover, encouraging results obtained in phase II clinical trial for SCCHN using a combination of monalizumab and cetuximab suggested that, targeting checkpoint receptors on NK cells, may be an efficient tool to complement first-generation immunotherapies against cancer (99).

Of notice is also the discovery of soluble forms of LAG-3 (sLAG3) and PD-1 (sPD-1) (70, 104). Different studies have been focused on the role of sPD-1 as a putative antitumor agent. Indeed, in mice an increase in anti-tumor activity was observed upon delivery of sPD-1 encoding plasmid at tumor site (105, 106). Moreover, clinical studies have investigated the presence of sPD-1 and its correlation with the overall survival of patients with different cancers (107, 108). It has been shown that sLAG3 is able to induce NK cytokines (IFN-γ and TNF-α) production in *ex vivo* assay (109). Moreover, a phase II clinical trial is investigating the role of sLAG3 in stimulating the immune system in combination with anti-PD-1 therapy (70).

## Conclusions and Future Perspectives

It is now evident that NK/ILC family plays a pivotal role in the immune defenses. Recent studies in murine and human settings demonstrated that the expression of several inhibitory checkpoints, that may be detrimental in the tumor context, is not restricted to T lymphocytes, revealing an important, yet poorly appreciated, contribution of their expression on innate immune cells. Thus, in the recent years different immunotherapy approaches, based on the blockade of inhibitory NK cell receptors, have been developed in order to unleash NK cell cytotoxicity. This is particularly important in the context of tumors that downregulate HLA-I expression and become invisible to T cells. However, it must be considered that most inhibitory checkpoints, targeted by mAbs therapies, are shared by T, NK and ILCs. Therefore, further studies are required in order to identify all the receptors regulating NK/ILC cells function for the development of new mAbs-based immunotherapies. In addition, considering the role exerted by the tumor microenvironment on ILCs plasticity and functions, it is necessary to better clarify the role of tumor infiltrating innate immune cells to improve the selectivity of cancer therapies. Therefore, also accurate patient analysis and deeper examinations of tumor biopsies will become key aspects to consider in order to construct personalized protocols. In this context, studies have been performed to determine the exact number of biopsies required to have a more precise PD-L1 expression profile that would more closely resemble to whole tumor section (110–112). Thus, despite the great improvement reached in the last years, further studies are required to investigate the expression of these checkpoints both in NK cells and on the other subsets of ILCs, and their precise role in human pathologies in order to improve the efficacy of immunotherapies thanks to a more personalized approach.

## Author Contributions

All authors listed have made a substantial, direct and intellectual contribution to the work, and approved it for publication.

### Conflict of Interest Statement

The authors declare that the research was conducted in the absence of any commercial or financial relationships that could be construed as a potential conflict of interest.
